# Dysentery

**Published:** 1876-06

**Authors:** 

**Affiliations:** Wiesbaden


### DYSENTERY.

By DR. CASPARIA, of Wiesbaden.

*Its treatment with Nitrate of Soda, (Saltpeter du Chile.)*—The Dr. learned to appreciate the value of this remedy in the winter of 1870–71. In Sept., 18 per cent., and in Oct., 30 per cent., of all patients entering the Frankfort Hospital, where he was on duty, had dysentery. He very justly remarks: "The result obtained in so great a number of cases are certainly conclusive as to the value of this agent in this affection." Dr. Widlinger, one of his colleagues at the hospital, who experimented with this drug, obtained like good results. During the violent epidemic which raged in 1822, the results obtained were astonishing, the mortality being scarcely 2 per cent. The Dr. states, that he has for a long time employed the nitrate of soda for both dysentery proper and the dysenteric diarrhœa. The dose must vary in these affections according to the degree of the phlegmasia.

The Dr. observes:—"In rectal dysentery, in a robust person, 25 grammes may be administered, in broken doses, in twenty-four hours. The medicament is dissolved in water, and given in a gummy solution. The dose oscillates between 15 and 25 grammes when there is no inflammatory complication of the small intestines. In light cases, improvement will follow in twenty-four hours; in severe cases, in two or three days. If there be no change within twenty-four hours, the dose should be increased. If the tenesmus has ceased, but symptoms of phlegmasia of the small intestines persist or supervene, the dose must be reduced to 8, or even 5 grammes (pro die)."

He quotes Rademacher as saying, the administration of the nitrate of Soda rapidly diminishes the abdominal pains and the number of stools; if when the tenesmus has ceased, an increase in the number of discharges should be remarked, it need cause no anxiety, as it is due to the prolonged use of the remedy, and will rapidly cease. Dr. C. says if the affection be, however, more particularly of the small intestines, the medicament must be given in smaller doses. Too large a dose will exaggerate the inflammation and the morbid manifestations. In these cases he generally begins with 6 grammes (pro die), in divided * doses, given in an oily emulsion. The medicine should be given warm, as cold is contrary to this affection, and causes an immediate increase in the number of stools. The medicine must be aided by strict attention to diet and hygiene. Patient must abstain from solid food some days after recovery. (*Cincinnati Lancet Observer*, Oct. 1875.

*Subcutaneous injection of Chloroform in the treatment Facial Neuralgia. By Geo. Wood, M. D., C. M.

